# Relationship between Anti–SARS-CoV-2 S Abs and IFN-λ3 Levels in the Administration of Oxygen following COVID-19 Vaccination

**DOI:** 10.4049/immunohorizons.2200093

**Published:** 2023-01-16

**Authors:** Yuichiro Takeshita, Yasuo To, Yusuke Kurosawa, Toru Kinouchi, Kota Tsuya, Yuji Tada, Kenji Tsushima

**Affiliations:** * Department of Pulmonary Medicine, International University of Health and Welfare Narita Hospital, Narita, Chiba, Japan;; † Division of Respiratory Medicine, Department of Internal Medicine, Nihon University School of Medicine, Itabashiku, Tokyo, Japan; and; ‡ Department of Respirology, Graduate School of Medicine, Chiba University, Chuo-ku, Chiba, Japan

## Abstract

Although the effectiveness of vaccination at preventing hospitalization and severe coronavirus disease (COVID-19) has been reported in numerous studies, the detailed mechanism of innate immunity occurring in host cells by breakthrough infection is unclear. One hundred forty-six patients were included in this study. To determine the effects of vaccination and past infection on innate immunity following SARS-CoV-2 infection, we analyzed the relationship between anti–SARS-CoV-2 S Abs and biomarkers associated with the deterioration of COVID-19 (IFN-λ3, C-reactive protein, lactate dehydrogenase, ferritin, procalcitonin, and D-dimer). Anti-S Abs were classified into two groups according to titer: high titer (≥250 U/ml) and low titer (<250 U/ml). A negative correlation was observed between anti–SARS-CoV-2 S Abs and IFN-λ3 levels (*r* = −0.437, *p* < 0.001). A low titer of anti–SARS-CoV-2 S Abs showed a significant association with oxygen demand in patients, excluding aspiration pneumonia. Finally, in a multivariate analysis, a low titer of anti–SARS-CoV-2 S Abs was an independent risk factor for oxygen demand, even after adjusting for age, sex, body mass index, aspiration pneumonia, and IFN-λ3 levels. In summary, measuring anti–SARS-CoV-2 S Abs and IFN-λ3 may have clinical significance for patients with COVID-19. To predict the oxygen demand of patients with COVID-19 after hospitalization, it is important to evaluate the computed tomography findings to determine whether the pneumonia is the result of COVID-19 or aspiration pneumonia.

## Introduction

On November 26, 2021, a new variant of the severe acute respiratory syndrome coronavirus 2 (SARS-CoV-2), known as the B.1.1.529 or ο variant, was first reported in South Africa to the World Health Organization Technical Advisory Group on SARS-CoV-2 Virus Evolution (1). This variant has the ability to circumvent immunity established by previous infections and vaccinations, resulting in a rapid increase in infections in the short term and breakthrough infections despite vaccination (2). Although adaptive immunity through vaccination alone cannot protect from breakthrough infection, studies have shown that vaccination is effective in preventing hospitalization from severe coronavirus disease (COVID-19) (3–6). However, the mechanism through which postvaccination infection or breakthrough infection affects innate immunity in host cells is unclear.

The titers of anti–SARS-CoV-2 S Abs correlate with neutralizing Ab production, which is a hallmark of adaptive immunity by vaccination (7–9). The spike protein (S protein) is located on the surface of SARS-CoV-2 and has two structures: S1 and S2. S1 consists of a receptor binding domain (RBD), which facilitates entry into the host cell by recognizing the angiotensin-converting enzyme 2 receptor on the cell. Because Abs that bind to RBD (anti–SARS-CoV-2 S Abs) are produced by mRNA vaccines, this immune mechanism can block the entry of SARS-CoV-2 into host cells (10–12). Also, an immune response to proteins generated in host cells based on mRNA information is established, and adaptive immunity, such as Ab production and cellular immunity, is established (13).

IFN-λ3 is a clinically useful marker that reflects innate immune activity following infection with viruses, including SARS-CoV-2 (14). However, few reports have shown the clinical usefulness of this biomarker in COVID-19. A previous report revealed that IFN-λ3 in the early phase of COVID-19 infection can predict the oxygen requirements of a patient (15).

With respect to the pathophysiology of COVID-19, no studies have focused on the relationship between anti–SARS-CoV-2 S Abs and IFN-λ3. Therefore, the aim of this study is to understand the effects of vaccination and past infections on innate immunity following SARS-CoV-2 infection.

## Materials and Methods

### Study design and patients

**FIGURE 1.**
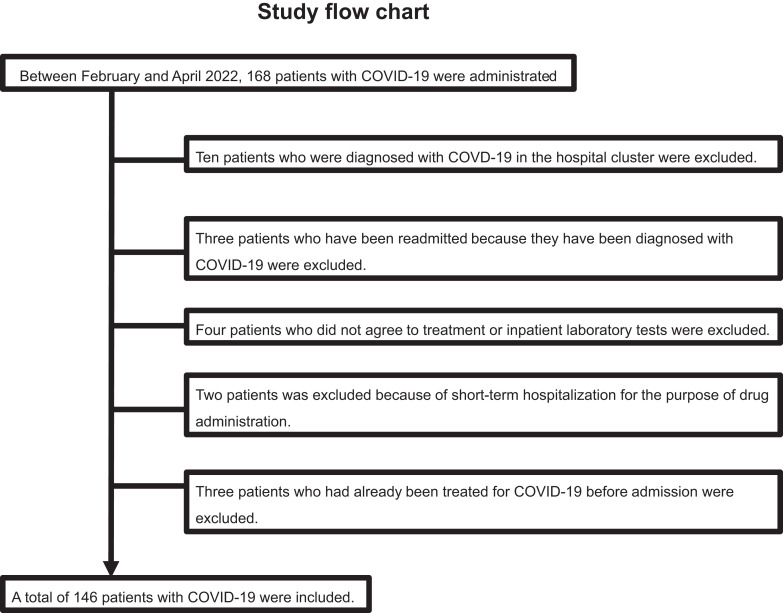
Study population flowchart. Between February and April 2022, 168 patients were administrated. Among these patients, 22 patients were excluded. As a result, 146 patients were enrolled in our study.

This single-center retrospective study evaluated 168 patients with COVID-19 who were admitted to the International University of Health and Welfare Narita Hospital between February and April 2022. COVID-19 infection was confirmed by quantitative RT-PCR. Among the 168 patients with COVID-19 admitted to our hospital during this period, 10 patients who were diagnosed with COVD-19 in the hospital cluster were excluded. Three patients who had been readmitted after being diagnosed with COVID-19 were excluded, 4 patients who did not agree to inpatient treatment or laboratory tests were excluded, 2 patients were excluded because of short-term hospitalization for the purpose of drug administration, and 3 patients who had already been treated for COVID-19 before admission were excluded. As a result, 146 patients were enrolled in our study. The study flowchart is shown in (Fig. 1.

### Clinical assessment

Data were extracted from the electronic medical records obtained for each patient upon hospitalization, which included their symptoms, vital signs, peripheral capillary oxygen saturation (SpO_2_), oxygen demand, laboratory test results, computed tomography (CT) scan results, age (in years), sex, and body mass index (BMI; in kg/m^2^). Blood tests were performed on day 1 of admission or 1 d before or after day 1 and were evaluated as laboratory findings on admission. The CT findings upon admission were focused on determining the presence of COVID-19 pneumonia and aspiration pneumonia, which was classified by two skilled operators (one radiologist and one pulmonologist) blinded to the clinical history.

### Definition of the disease severity

According to the Ministry of Health, Labor and Welfare of Japan, disease severity was categorized into four stages: mild, moderate I, moderate II, and severe. Mild disease was defined as a lack of respiratory symptoms, pneumonia, and oxygen saturation levels (SpO_2_) ≥96%. Moderate disease I was defined as mild respiratory symptoms, radiological pneumonia findings, and 93% < SpO_2_ < 96%. Moderate disease II was defined as SpO_2_ ≤93% requiring oxygen support. Severe disease was defined as requiring mechanical ventilation (MV) or extracorporeal membrane oxygenation support for acute respiratory distress syndrome (16).

### Quantitative measurement method of anti-S Abs

Anti–SARS-CoV-2 S Abs were measured using the Elecsys anti–SARS-CoV-2 S immunoassay, which is a quantitative electrochemiluminescence immunoassay that detects Abs to the SARS-CoV-2 S protein RBD. As a result of the automatic analysis in this assay, ≥0.80 U/ml was interpreted as positive and <0.80 U/ml was considered negative for anti–SARS-CoV-2 S Abs (17, 18). Although the values between 0.40 and 250 U/ml exhibited a linearity, the results below this range were set to 0.4 U/ml. The samples above 250 U/ml were diluted into the linear range of the assay (1:10 or 1:100) with Diluent Universal reagent (Roche Diagnostics, Rotkreuz, Switzerland). Therefore, the upper limit value that could be measured within this range was 25,000 U/ml (9).

### Statistical analysis

Summary statistics were calculated using the mean ± SD, frequency distributions, or proportions for baseline variables. We first compared the mean values (±SD) and quartiles between the two groups for continuous variables. Subsequently, the Kolmogorov-Smirnov test (two-sided) and Shapiro-Wilk test were used to test normality, after which homoscedasticity was determinedusing the *F*-test. Next, the Welch *t* test and Mann-Whitney *U* test were performed according to the data distribution. For continuous variables, such as age and BMI, we first compared the mean values (±SD) and quartiles between the two groups. Then, Fisher’s exact test was used to determine the significance of the differences based on the groups. Key characteristics of the variables were later evaluated. For the analysis of 146 patients, a logistic regression model was fitted with age, male sex, BMI, aspiration pneumonia, and anti-S Ab <250 U/ml. A *p* value <0.05 was considered statistically significant. Cutoff values were also evaluated using a receiver operator characteristic (ROC) curve analysis and an area under the ROC curve (AUC). Higher AUC values demonstrated superior discriminatory ability as follows: excellent discrimination, 0.9 ≤ AUC; good discrimination, 0.80 ≤ AUC < 0.90; fair discrimination, 0.70 ≤ AUC < 0.80; and poor discrimination, AUC <0.70. For a diagnostic test to be meaningful, the AUC must be >0.5 (19, 20). Outliers for each variable were identified by the Smirnov-Grubbs test. Finally, all statistical analyses were conducted using EZR (Saitama Medical Center, Jichi Medical University, Saitama, Japan), a graphical user interface for R, and a modified version of the R commander designed to add statistical functions that are frequently used in biostatistics (21).

## Results

### Background of the patients

Table I shows the clinical characteristics of the 146 patients enrolled in this study by comparing the oxygen-nonrequiring group (*n* = 78) with the oxygen-requiring group (*n* = 68). Based on a univariate analysis, the percentage of hypertension, pneumonia upon admission, aspiration pneumonia upon admission, and do not intubate cases, as well as the values for age, C-reactive protein, lactate dehydrogenase, ferritin, IFN-λ3, and D-dimer, were significantly higher in the oxygen-requiring group than in the oxygen-nonrequiring group.

**FIGURE 2.**
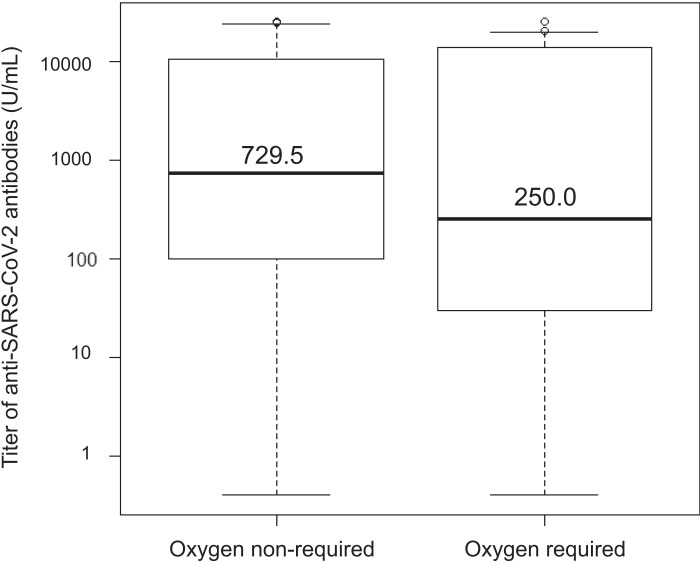
Box plot showing the titers of anti–SARS-CoV-2 S Abs, comparing the oxygen-nonrequiring group with the oxygen-requiring group. The bottom and top of the box show the 25th and 75th percentile values. The horizontal line in the box represents the median. The median titers of anti–SARS-CoV-2 S Abs in the oxygen-nonrequiring group was 729.5 U/ml, the 25th percentile value was 105.6 U/ml, and the 75th percentile value was 10,048.0 U/ml. The median titers of anti–SARS-CoV-2 S Abs in the oxygen-requiring group was 250.0 U/ml, the 25th percentile value was 30.2 U/ml, and the 75th percentile value was 13,662.0 U/ml. The horizontal line under the whisker extending farther down from the bottom of the box to the bottom represents the 10th percentile value, and the horizontal line above the whisker extending farther up from the top of the box represents the 90th percentile value.

**Table I.** Characteristics and clinical outcomes of all patients

Variables	All patients (*n* = 146)	Oxygen administration		
		Nonrequired (*n* = 78)	Required (*n* = 68)	*p* Values
Age, y	70.2 ± 18.7	62.6 ± 20.9	79.0 ± 10.1	<0.0001
Male sex, %	94 (64.4%)	45 (57.7%)	49 (72.1%)	0.0842
BMI, kg/m^2^	24.69 ± 5.59, NA = 37	25.31 ± 5.49, NA = 5	23.45 ± 5.65, NA = 32	0.1020
Hypertension, %	69 (47.3%)	29 (37.2%)	40 (58.9%)	0.0125
Diabetes mellitus, %	26 (17.8%)	15 (19.2%)	11 (16.2%)	0.6700
Dyslipidemia, %	29 (19.9%)	16 (20.5%)	13 (19.1%)	>0.9999
History of vaccination two times or more, %	68 (78.2%), NA = 59	38 (73.7%), NA = 26	30 (85.7%), NA = 33	0.1940
Duration from symptom onset to admission, d	3.5 ± 3.4, NA = 12	3.2 ± 3.2, NA = 5	3.9 ± 3.5, NA = 7	0.2450
CRP, mg/dl	6.96 ± 8.04	3.72 ± 4.62	10.67 ± 9.44	<0.0001
LDH, U/L	294 ± 182, NA = 7	246 ± 67, NA = 4	349 ± 245, NA = 3	0.0016
Ferritin, ng/ml	375 ± 421, NA = 13	245 ± 243, NA = 10	512 ± 516, NA = 3	0.0003
IFN-λ3, pg/ml	8.4 ± 11.2, NA = 21	6.3 ± 5.9, NA = 14	10.6 ± 14.5, NA = 7	0.0349
PCT, ng/ml	1.84 ± 10.49, NA = 15	0.23 ± 0.62, NA = 11	3.52 ± 14.87, NA = 4	0.0817
d-dimer, μg/ml	3.39 ± 8.52, NA = 4	1.54 ± 2.40, NA = 3	5.47 ± 11.84, NA = 1	0.0094
Anti–SARS-CoV-2 S Abs, U/ml	424.0, (66.0, 12,019.0), NA = 57	729.5, (105.6, 10,048.0), NA = 34	250.0, (30.2, 13,662.0), NA = 23	0.9950
Anti–SARS-CoV-2 Abs <250 U/ml	32 (36.0%), NA = 57	16 (36.4%), NA = 34	22 (48.9%), NA = 23	0.2860
Pneumonia on admission, %	88 (60.3%)	31 (39.7%)	57 (83.8%)	<0.0001
Aspiration pneumonia on admission, %	19 (13.0%)	4 (5.1%)	15 (22.1%)	0.0029
DNI, %	38 (26.0%)	5 (6.4%)	33 (48.5%)	<0.0001

Data are presented as the mean ± SD, median (interquartile range), or *n* (%). CRP, C-reactive protein; DNI, do not intubate; LDH, lactate dehydrogenase; NA, not available; PCT, procalcitonin.

### Definition of high and low titers of anti–SARS-CoV-2 S Abs

(Fig. 2 shows the distribution of anti–SARS-CoV-2 S Abs divided into two groups: patients who required oxygen administration after hospitalization, an oxygen-requiring group; and patients who did not require oxygen administration after hospitalization, an oxygen-nonrequiring group. Although no statistically significant difference in anti–SARS-CoV-2 S Abs was observed between the two groups (shown in Table I), the box plot shown in (Fig. 2 indicates that the median and 25th percentile titers of anti–SARS-CoV-2 S Abs in the oxygen-requiring group were lower than those in the oxygen-nonrequiring group. Visually, the titer of anti–SARS-CoV-2 S Abs in the oxygen-requiring group was lower than that in the oxygen-nonrequiring group. Furthermore, ROC analysis of the anti-S Abs, which were predicted to account for oxygen administration, revealed that the cutoff value for the anti-S Ab titer was 250 U/ml (AUC, 0.537; specificity, 61.4%; sensitivity, 53.3%; 95% confidence interval, 0.415–0.658; shown in Supplemental Fig. 1). Therefore, a titer of 250 U/ml or more was defined as a high titer of anti-S Abs; otherwise, a low titer of anti-S Abs was indicated.

**FIGURE 3.**
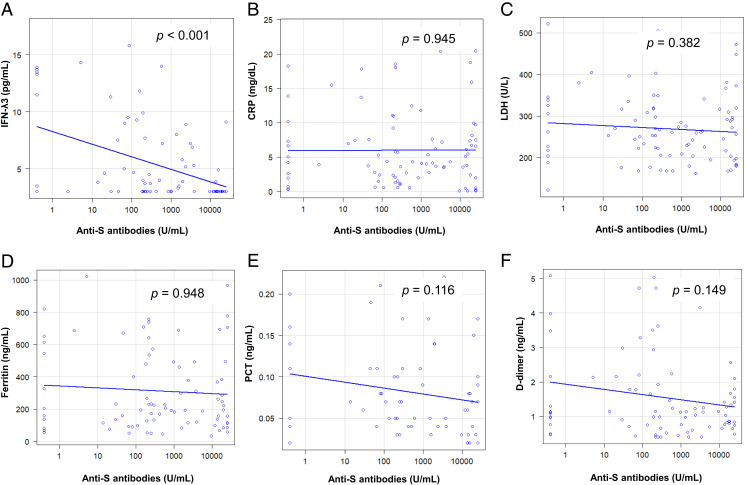
Correlation between anti–SARS-CoV-2 S Abs and COVID-19 severity prediction markers. Correlation between anti-SARS-CoV-2 S Abs and each COVID-19 severity prediction marker [(**A**) IFN-λ3; (**B**) CRP; (**C**) LDH; (**D**) Ferritin; (**E**) PCT; (**F**) D-dimer] is shown. A significantly negative correlation was observed between a value of IFN-λ3 and the titer of anti-S Abs (*p* < 0.0001, *r* = −0.437). The outliers for each variable were excluded and are shown in the graph. CRP, C-reactive protein; LDH, lactate dehydrogenase; PCT, procalcitonin.

### Differences in backgrounds and clinical outcomes between high titer of anti–SARS-CoV-2 S Abs group and low titer of SARS-CoV-2 S Abs group

Table II lists the differences in backgrounds and clinical outcomes between the high-titer anti–SARS-CoV-2 S Ab group (≥250 U/ml) and the low-titer anti–SARS-CoV-2 S Ab group (<250 U/ml). Because anti–SARS-CoV-2 S Abs were not available in 57 patients, the analysis was done with the remaining 89 patients. On the basis of univariate analysis, although a significantly higher proportion of dyslipidemia was observed in the high-titer anti–SARS-CoV-2 S Ab group, the IFN-λ3 value and the proportion of pneumonia were significantly higher in the high-titer anti–SARS-CoV-2 S Ab group.

**Table II.** Differences in backgrounds and clinical outcomes between the high-titer anti-S Ab group and the low-titer anti-S Ab group

Variables	Anti–SARS-CoV-2 Abs <250 U/ml (*n* = 38)	Anti–SARS-CoV-2 Abs ≥250 U/ml (*n* = 51)	*p* Values
Age, y	74.8 ± 12.9	71.1 ± 19.4	0.2820
Male sex, *n* (%)	27 (71.1%)	36 (70.6%)	>0.9999
BMI, kg/m^2^	23.19 ± 4.40, NA = 9	25.33 ± 5.00, NA = 17	0.0785
Hypertension, *n* (%)	18 (47.4%)	25 (49.0%)	>0.9999
Diabetes mellitus, *n* (%)	6 (15.8%)	7 (13.7%)	>0.9999
Dyslipidemia, *n* (%)	2 (5.3%)	13 (25.5%)	0.0197
History of vaccination two times or more, *n* (%)	14 (77.8%), NA = 20	29 (93.5%), NA = 20	0.1750
Duration from symptom onset to admission, d	3.4 ± 3.5, NA = 4	4.3 ± 3.3, NA = 3	0.2960
CRP, mg/dl	7.43 ± 7.54	7.21 ± 8.70	0.9030
LDH, U/L	301 ± 113	291 ± 239	0.8010
Ferritin, ng/ml	440 ± 423	385 ± 477	0.5800
IFN-λ3, pg/ml	11.7 ± 16.5	5.4 ± 5.5	0.0282
PCT, ng/ml	1.12 ± 4.68, NA = 1	2.01 ± 12.35	0.6390
d-dimer, μg/ml	2.79 ± 3.33	3.20 ± 8.72	0.7600
DNI, *n* (%)	9 (23.7%)	13 (25.5%)	>0.9999
Pneumonia, %	30 (30/38)	128 (28/51)	0.0246
Aspiration pneumonia, %	5 (5/38)	8 (8/51)	>0.9999

Data are presented as the mean ± SD, median [interquartile range], or n (%).CRP, C-reactive protein; DNI, do not intubate; LDH, lactate dehydrogenase; NA, not available; PCT, procalcitonin.

### Clinical outcome of severity by comparing the high- and low-titer anti–SARS-CoV-2 S Ab groups

The clinical outcomes of severity are listed in Tables III and IV. As clinical outcomes, severity upon admission (mild, moderate I, moderate II, and severe) and oxygen demand during hospitalization were included. The analyses were divided into two groups: a high-titer anti–SARS-CoV-2 S Ab group (≥250 U/ml) and a low-titer SARS-CoV-2 S Ab group (<250 U/ml). Table III presents the analysis for all patients, including those with aspiration pneumonia. No statistically significant differences were observed between the two groups. Table IV shows the results for the analysis of patients excluding aspiration pneumonia. Compared with the high-titer anti–SARS-CoV-2 S Ab group, the proportion of oxygen demand during hospitalization was significantly higher in the low-titer group. Although no statistically significant difference was observed, the percentage of mild patients upon admission tended to be higher in the high-titer group than in the low-titer group.

**Table III.** Clinical outcomes of severity in all patients

Clinical outcomes	All patients (*n* = 146)	Anti–SARS-CoV-2 Abs <250 U/ml (*n* = 38)	Anti–SARS-CoV-2 Abs ≥250 U/ml (*n* = 51)	*p* Values
Severity on admission				
Mild, *n* (%)	51 (34.9%)	8 (21.1%)	19 (37.3%)	0.1100
Moderate I, *n* (%)	36 (24.7%)	11 (28.9%)	11 (21.6%)	0.4640
Moderate II, *n* (%)	54 (37.0%)	16 (42.1%)	20 (39.2%)	0.8290
Severe, *n* (%)	4 (2.7%)	2 (5.3%)	1 (2.0%)	0.5730
Oxygen demand during hospitalization, *n* (%)	68 (46.6%)	22 (57.9%)	23 (45.1%)	0.2860
Lower level of oxygen demand, *n* (%)	31 (21.2%)	10 (26.3%)	10 (19.6%)	0.6080
Higher level of oxygen demand, *n* (%)	22 (15.1%)	8 (21.1%)	10 (19.6%)	>0.9999
Mechanical ventilation, *n* (%)	1 (0.7%)	1 (2.6%)	0 (0%)	0.4270
Death, *n* (%)	14 (9.6%)	3 (7.9%)	3 (5.9%)	>0.9999

Lower level of oxygen demand was defined as oxygen administration <4 L/min; higher level of oxygen demand was defined as oxygen administration ≥5 L/min or high-flow oxygen therapy.

**Table IV.** Clinical outcomes of severity in patients without aspiration pneumonia

Clinical outcomes	All patients (*n* = 127)	Anti–SARS-CoV-2 Abs <250 U/ml (*n* = 33)	Anti–SARS-CoV-2 Abs ≥250 U/ml (*n* = 43)	*p* Values
Severity on admission				
Mild, *n* (%)	50 (39.4%)	7 (21.2%)	19 (44.2%)	0.0511
Moderate I, *n* (%)	32 (25.2%)	9 (27.3%)	11 (25.6%)	>0.9999
Moderate II, *n* (%)	40 (31.5%)	14 (42.4%)	12 (27.9%)	0.2270
Severe, *n* (%)	4 (3.1%)	2 (6.1%)	1 (2.3%)	0.5760
Oxygen demand during hospitalization, *n* (%)	53 (41.7%)	20 (60.6%)	15 (34.9%)	0.0367
Lower level of oxygen demand, *n* (%)	25 (19.7%)	10 (30.3%)	6 (14.0%)	0.0968
Higher level of oxygen demand, *n* (%)	18 (14.2%)	7 (21.2%)	7 (16.3%)	0.7660
Mechanical ventilation, *n* (%)	1 (0.8%)	1 (3.0%)	0 (0%)	0.4340
Death, *n* (%)	9 (7.1%)	2 (6.1%)	2 (4.7%)	>0.9999

Lower level of oxygen demand was defined as oxygen administration <4 L/min; higher level of oxygen demand was defined as oxygen administration ≥5 L/min or high-flow oxygen therapy.

### Correlation between anti-S Abs and COVID-19 severity prediction markers

(Fig. 3 shows the correlations between anti–SARS-CoV-2 S Abs and COVID-19 severity prediction markers. On the basis of previous reports, IFN-λ3, C-reactive protein, lactate dehydrogenase, ferritin, procalcitonin, and D-dimer were considered predictive markers. *p* Values were calculated using the Spearman rank-order correlation test. A negative correlation was observed between anti–SARS-CoV-2 S Abs and IFN-λ3 levels (*r* = −0.437, *p* < 0.001). No significant correlation was observed between anti–SARS-CoV-2 S Abs and the other predictive markers.

### Multivariate logistic regression analysis of factors for oxygen demand

Table V lists the factors accounting for oxygen demand. Factors including age, male sex, BMI, aspiration pneumonia, IFN-λ3, and anti-S Abs <250 U/ml were analyzed. A multivariate analysis indicated that anti-S Abs <250 U/ml significantly affected oxygen demand (odds ratio, 3.670; 95% confidence interval, 1.040–12.900; *p* = 0.0426).

**Table V.** Multivariate logistic regression analysis of factors for oxygen demand during hospitalization

Variables	Oxygen demand		
	Odds ratio	95% CI	*p* Values
Age y	1.040	0.997–1.090	0.0640
Male sex, %	3.350	0.860–13.100	0.0814
BMI, kg/m^2^	1.110	0.965–1.280	0.1420
Aspiration pneumonia, %	7.390	0.525–104.000	0.1380
IFN-λ3, pg/ml	0.987	0.879–1.110	0.8280
Anti–SARS-CoV-2 S Abs <250 U/ml	3.670	1.040–12.900	0.0426

CI, confidence interval.

## Discussion

Our study revealed several findings. First, a univariate analysis revealed that patients with low-titer anti–SARS-CoV-2 S Abs, which reflects low production of neutralizing Abs, exhibited a significant association with IFN-λ3 levels and pneumonia. Furthermore, analysis of the correlation between the titer of anti–SARS-CoV-2 S Abs and markers of COVID-19 severity showed an inverse correlation between the titer of anti–SARS-CoV-2 S Abs and the IFN-λ3 levels. Second, in the analysis of patients, excluding aspiration pneumonia, patients with a low titer of anti–SARS-CoV-2 S Abs showed a significant association with oxygen demand. Third, in a multivariate analysis, a low titer of anti–SARS-CoV-2 S Abs was an independent risk factor for oxygen demand even after adjusting for age, sex, BMI, aspiration pneumonia, and IFN-λ3 levels.

Low titer of anti–SARS-CoV-2 S Abs may affect COVID-19 severity. Kurano et al. reported that patients receiving MV had a weaker response of the IgG S1 protein and IgG RBD, which are considered neutralizing Abs, than those who did not require MV (22). Moreover, our results indicated that the low-titer anti–SARS-CoV-2 S Ab group significantly affected oxygen demand compared with the high-titer group. Thus, sufficient amounts of neutralizing Abs may prevent the severity of COVID-19 following vaccination. As a point of caution, it has been noted that patients with immunosuppressive conditions such as dialysis, organ transplant, and blood tumors have a weak Ab response to the vaccine (23–25). Therefore, measuring anti–SARS-CoV-2 S Abs may be useful for predicting severe COVID-19.

Although a lower titer of anti–SARS-CoV-2 S Abs had no significant effect on oxygen demand during hospitalization for all patients, it was associated with oxygen demand when aspiration pneumonia was excluded. One reason for the difference may be that the mean age of the patients included in this study was 70.2 ± 18.7 y, which was considered elderly. Elderly patients infected with SARS-CoV-2 are more likely to be frail, which may lead to aspiration pneumonia and a greater need for oxygen (26, 27). Because it can also affect the type of treatment, such as antibiotic administration, it is important to have skilled radiologists to interpret the chest CT to differentiate between COVID-19 pneumonia and aspiration pneumonia.

In the present study, the titer of anti–SARS-CoV-2 S Abs was inversely associated with IFN-λ3 values, which indicates clinical significance. As mentioned above, a high anti–SARS-CoV-2 S Ab titer has the ability to neutralize SARS-CoV-2, blocking the virus entry into host cells. Thus, even if viral infection is established, its ability to invade host cells may be ameliorated by neutralizing Abs. This may also prevent subsequent innate and acquired immunity, which leads to severe cases of COVID-19. However, IFN is a cytokine that may be divided into three major classes: type I (IFN-α, β), type II (IFN-γ), and type III (IFN-λ). These IFNs are inhibitors of viral infection during the innate immune system response and function as the first line of defense against pathogens, including SARS-CoV-2 (28–30). The IFN-λs are a family of innate immune cytokines consisting of IFN-λs 1–4 that are important mediators of barrier immunity (31). Sugiyama et al. previously demonstrated that increases in IFN-λ3 concentrations in the serum of patients with COVID-19 is predictive and occurs a few days before oxygen administration is necessary (15). The inverse relationship between the value of IFN-λ3 and the titer of anti–SARS-CoV-2 S Abs suggests that the strong neutralizing ability may minimize SARS-CoV-2 infection, which may reduce the subsequent activation of innate immunity. Furthermore, the correlation between anti–SARS-CoV-2 S Abs and IFN-λ3 was not significantly different when analyzed in 19 patients with aspiration pneumonia (*r* = −0.43, *p* = 0.1420), whereas there was a significant difference when 127 patients with aspiration pneumonia were excluded (*r* = −0.445, *p* < 0.0001). One of the reasons for this may be that there were few patients (*n* = 19) with aspiration pneumonia in the present study. Further studies are needed to verify whether COVID-19 with aspiration pneumonia alters the association between anti–SARS-Cov-2 S Abs and IFN-λ3.

Our study had several limitations. First, this study was a single-center study. Second, the overall sample size for the final cohort was limited; however, the frequency of vaccination, the virus variant, and the degree of severity can change, depending on which parameter is the subject of the study. Therefore, the time frame of the study was limited to February to April 2022, which was the sixth wave in Japan (32). Third, the AUC of the anti-S Ab titer showed poor discrimination (AUC, 0.537) by ROC analysis. One reason may be that the mean titer of the anti–SARS-CoV-2 S Abs in all patients was 5,915.9 U/ml with an SD of 8,845.3 U/ml, which indicated high variability. Further studies are needed to determine the cutoff value for the anti–SARS-CoV-2 S Abs. Fourth, although several potential biomarkers were examined for severity in correlation to the anti–SARS-CoV-2 S Abs, only IFN-λ3 was included as an early innate immunity. Indeed, it may be necessary to show that IFN-λ3 is more associated with the anti–SARS-CoV-2 S Abs than are other innate immune-related IFNs. IFN-λ3 has been the only innate immunity-related marker that is covered by insurance for patients with COVID-19 in Japan and can be measured quickly within our facility. To support the result of our study, further research is required on the association between innate immune markers and the anti–SARS-CoV-2 S Abs. Fifth, it is unclear if this low Ab population is the result of low vaccination; poor immunity; or, given that the present study was performed in the sixth wave of COVID-19 surges in Japan, the Ab methodology is just not picking up the later variants. However, because it is well known that infection drives a broader Ab repertoire, it is plausible that the patients with low Ab titers represented in this study may have functional immunity. Further research is required validating the low-Ab population by a second Ab validation method such as a neutralization assay with live or pseudotyped virus. Sixth, male sex, diabetes, vaccination history, and BMI did not correlate with disease severity, because all of these have been shown to affect outcomes. This may indicate that the cohort size is too small or that some unknown selection bias has been introduced. Finally, in clinical practice, it is sometimes difficult to distinguish whether pneumonia appearing in CT scans is characteristic of COVID-19 pneumonia or aspiration pneumonia. The knowledge of the patient’s preexisting conditions and general and mental status may help in the differential diagnosis (33). Therefore, it is important to comprehensively evaluate not only CT findings but also patient background and clinical symptoms to distinguish between COVID-19 and aspiration pneumonia.

In conclusion, measuring anti-S Abs, a biomarker for predicting the neutralization capacity following vaccination, and IFN-λ3, a biomarker for determining innate immunity activation resulting from SARS-CoV-2 infection, may have clinical implications for patients with COVID-19. Furthermore, determining whether patients have COVID-19 pneumonia or aspiration pneumonia may be important in determining the treatment regimen and can predict the severity after hospitalization. These data provide important information that warrant a prospective study on a larger scale in the future.

## Ethical approval

All study procedures were conducted according to the standards of the ethical review board of the International University of Health and Welfare (approval number 20-Nr-101; approved February 22, 2021) and conformed to the 1964 Declaration of Helsinki and its subsequent amendments or comparable ethical standards. The ethics committee waived the requirement for informed consent because this was a retrospective analysis limited to preexisting data, which were collected as part of the standard of care by respiratory physicians. Furthermore, data anonymization and privacy were protected.

## Supplementary Material

Supplemental Figure 1 (PDF)
