# Supplementary material for: Relationship between Anti–SARS-CoV-2 S Abs and IFN-λ3 Levels in the Administration of Oxygen following COVID-19 Vaccination
Source: Immunohorizons. 2023 Jan 16;7(1):97–105. doi: 10.4049/immunohorizons.2200093 (PMC10563441; doi:10.4049/immunohorizons.2200093)
Supplement: Supplemental Figure 1 (PDF) [file IH_2200093_Supplemental_1.pdf]

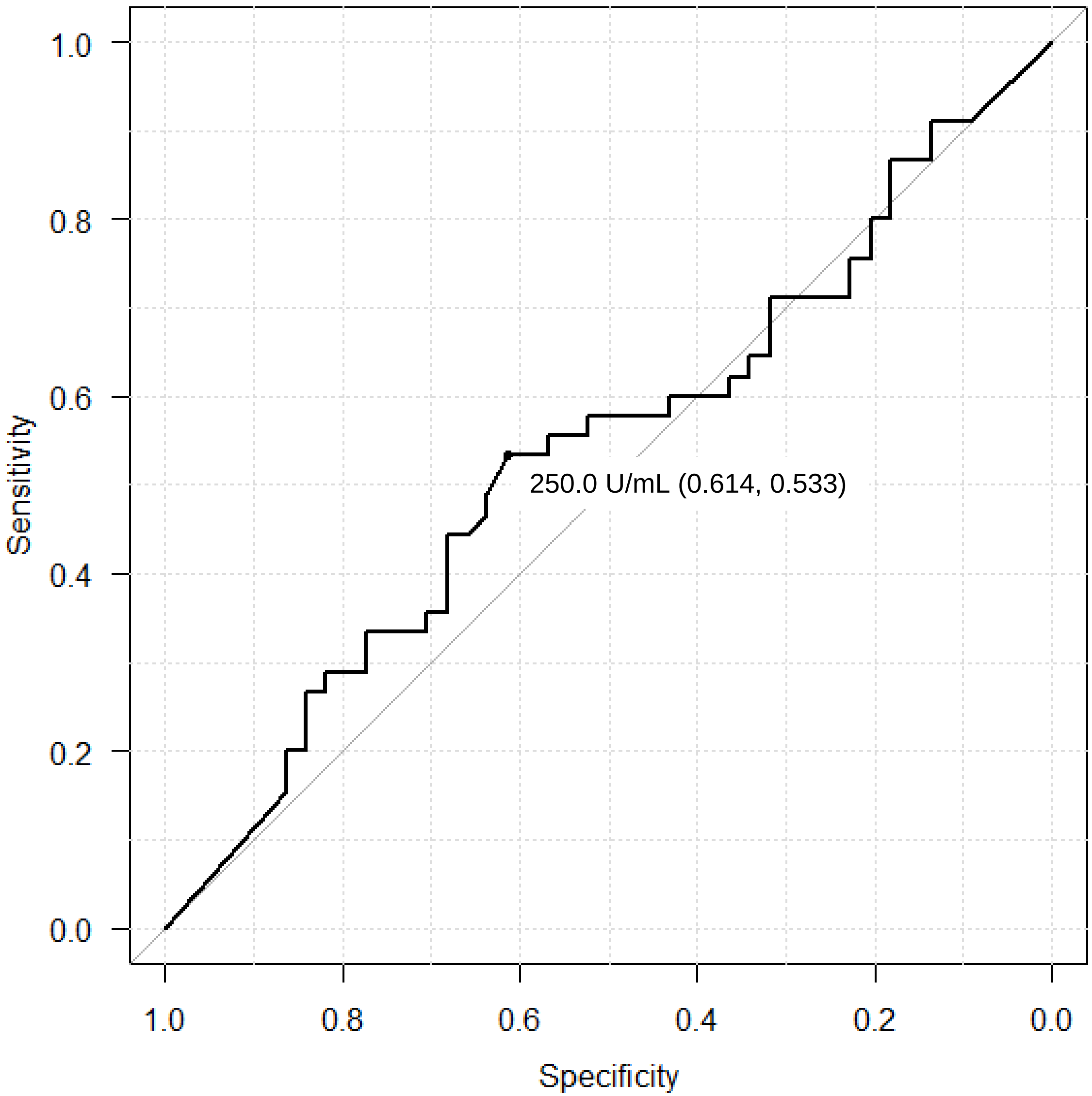

Supplemental Figure 1: ROC analysis of the anti-SARS-Cov-2 S antibodies, predicting oxygen administration. ROC, receiver operating characteristic.
